# Determination of significant parameters in remote ischemic postconditioning for ischemic stroke in experimental models: A systematic review and meta‐analysis study

**DOI:** 10.1111/cns.13925

**Published:** 2022-07-27

**Authors:** Kezhou Liu, Zhengting Cai, Quanwei Zhang, Jiatong He, Yinuo Cheng, Shaonong Wei, Mengjie Yin

**Affiliations:** ^1^ Department of Biomedical Engineering, School of Automation (Artificial Intelligence) Hangzhou Dianzi University Hangzhou China; ^2^ School of Management Hangzhou Dianzi University Hangzhou China; ^3^ HDU‐ITMO Joint Institute Hangzhou Dianzi University Hangzhou China

**Keywords:** experimental models, ischemia, meta‐analysis, remote ischemic postconditioning, stroke

## Abstract

**Objectives:**

To systematically review studies using remote ischemia postconditioning (RIPostC) for ischemic stroke in experimental models and obtain factors that significantly influence treatment outcomes.

**Materials and Methods:**

Peer‐reviewed studies were identified and selected based on the eligibility criteria, followed by extraction of data on potentially influential factors related to model preparation, postconditioning, and measure time based on outcome measures including infarct size, neurological scales, and cell tests with autophagy, apoptosis, normal‐neuron, and damaged‐neuron counting. Then, all data were preprocessed, grouped, and meta‐analyzed with the indicator of the standardized mean difference.

**Results:**

Fifty‐seven studies with 224 experiments (91 for infarct size, 92 for neurological scales, and 41 for cell‐level tests) were included. There was little statistical difference between different model preparations, treated body parts, number of treatments, and sides. And treatment effect was generally a positive correlation with the duration of conditioning time to stroke onset with exceptions at some time points. Based on infarct size, the number of cycles per treatment, duration of occlusion, and release per cycle showed significant differences. Combined with the effect sizes by other measures, the occlusion/release duration of 8–10 min per cycle is better than 5 min, and three cycles per treatment were most frequently used with good effects. Effect also varied when measuring at different times, showing statistical differences in infarct size and most neurological scales. RIPostC is confirmed as an effective therapeutic intervention for ischemic stroke, while the RIPostC‐mediated autophagy level being activated or inhibited remained conflicting.

**Conclusions:**

Conditioning time, number of cycles per treatment, duration of occlusion, and release per cycle were found to influence the treatment effects of RIPostC significantly. More studies on the relevant influential factors and autophagy mechanisms are warranted.

## Graphical abstract

Studies on remote ischemia postconditioning (RIPostC) for ischemic stroke have been conducted in experimental models for over a decade, but lacking a systematic review makes it unclear which combination of conditioning parameters achieves better efficacy and which measures better reflect treatment effect and disease progression. Our study focused on the therapeutic effect of RIPostC on acute ischemic stroke based on experimental models, using meta‐analysis to quantitatively assess as many potential influencing factors as possible by various outcome measures.

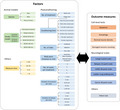

## 1 INTRODUCTION

The strategy of ischemic postconditioning (IPostC) initially originated in the field of cardiovascular diseases^1^ was then successfully applied to other clinical areas including liver^2^ and cerebral disorders.^3^ Studies have suggested that IPostC, consisting of several short, nonlethal periods of ischemia and reperfusion following an ischemia event, has a protective effect on multi‐organs^4^ by reducing the ischemia/reperfusion (I/R) injury induced by organ transplantation^5^ or vascular occlusion.^3^, ^6^ In addition to IPostC, there are also ischemic preconditioning (IPreC, performed prior to ischemia in an organ) and ischemic perconditioning (IPerC, performed during an ischemia event). And IPostC can be further divided into in situ ischemic postconditioning (ISIPostC) and remote ischemic postconditioning (RIPostC) depending on the site of intervention.^7^ All kinds of above‐mentioned conditioning methods play their respective parts in studying endogenous survival and protection mechanisms of multi‐organs involving multiple processes, but RIPostC has its own distinct strengths over others in the field of ischemic stroke. Firstly, RIPostC is considered as a feasible and promising therapeutic intervention in stroke, not just for mechanism research. In contrast, since the occurrence of stroke is unpredictable, IPreC has the issue of timeliness, which makes it hard to be a viable approach but may be used for the prevention of stroke in high‐risk groups.^8^ Secondly, the intervention site of RIPostC is usually selected on the limb, also known as remote limb ischemic postconditioning (RLIPostC), which owns the advantages of noninvasion or less invasion, inexpensiveness, and convenience.^4^

The research on RIPostC for ischemic stroke has been conducted in experimental models for over a decade, accumulating a large number of published scientific articles,^3^, ^6^, ^9^ and some institutions even conducted the clinical randomized trials.^10^, ^11^ However, studies designed different schemes of RIPostC and utilized various outcome measures according to their research objectives. This results in the reliability and credibility of the conclusions drawn from individual original research articles only with a few animals are limited, making it difficult to translate into valid guidance for clinical practice. At present, the absence of a systematic analysis and summary of these articles makes it unclear which RIPostC options are most effective for stroke recovery and which measures better reflect treatment effect and disease progression. Weir et al.^9^ have meta‐analyzed the relevant literatures on remote ischemic conditioning in experimental stroke, yet the content which was relatively general and poorly targeted containing not only IPostC but also IPreC. The inclusion of factors likely to impact the effects of RIPostC in their study was not all‐sided enough, and effect sizes were calculated based on common measures, without consideration of conditioning time, measure time, and some cell‐level tests like apoptosis and autophagy.

Our study mainly focused on the therapeutic effect of RIPostC on acute ischemic stroke based on experimental models, using meta‐analysis approaches to quantitatively assess as many potential influencing factors as possible by multidimensional outcome measures including three aspects. Besides the commonly used infarct size and neurological scales, the measure indicators particularly added some cell‐level tests including normal‐neuron density, damaged‐neuron counting, autophagy, and apoptosis. Nevertheless, since neuron death involves multiple sophisticated and interacted cell signaling pathways, with the associated mechanisms not fully understood,^3^, ^7^ the most symbolic biomarkers and detection methods were selected in the study rather than pathway‐specific molecules or factors for statistics.

## 2 MATERIALS AND METHODS

### 2.1 Search strategy

Peer‐reviewed research articles published before January 2021 were searched in PubMed, Web of Science, and Google Scholar for English language, with CNKI, CQVIP, and Wanfang for Chinese language. Search keywords contained three aspects, including stroke (“stroke” or “acute stroke” or “apoplexy” or “ischemia” or “cerebral ischemia” or “cerebrovascular accident”), experimental model (“model” or “experimental model” or “animal”), and remote ischemic postconditioning (“RIP” or “RIPerC” or “RIPostC” or “remote ischemic postconditioning” or “remote ischemic perconditioning”), and search terms were adjusted for the different search engines. We also consulted reference lists of relevant reviews and studies to identify additional articles.

### 2.2 Study selection

**FIGURE 1:**
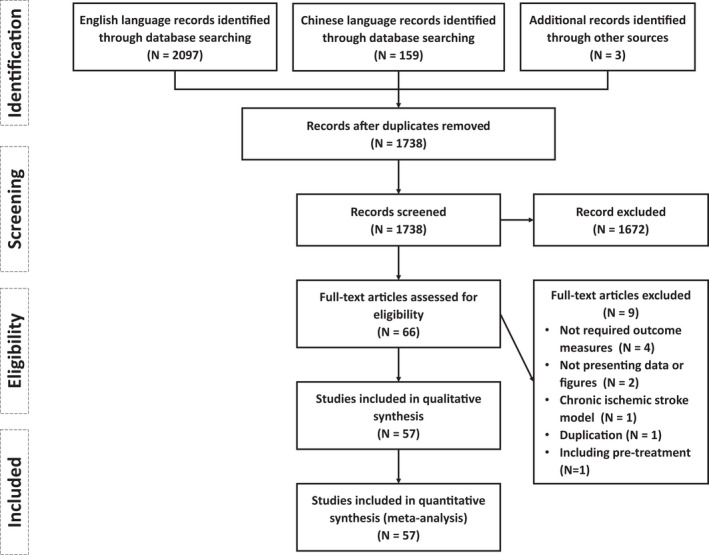
Flow chart of study and information through the different phases based on PRISMA Statement

**FIGURE 2:**
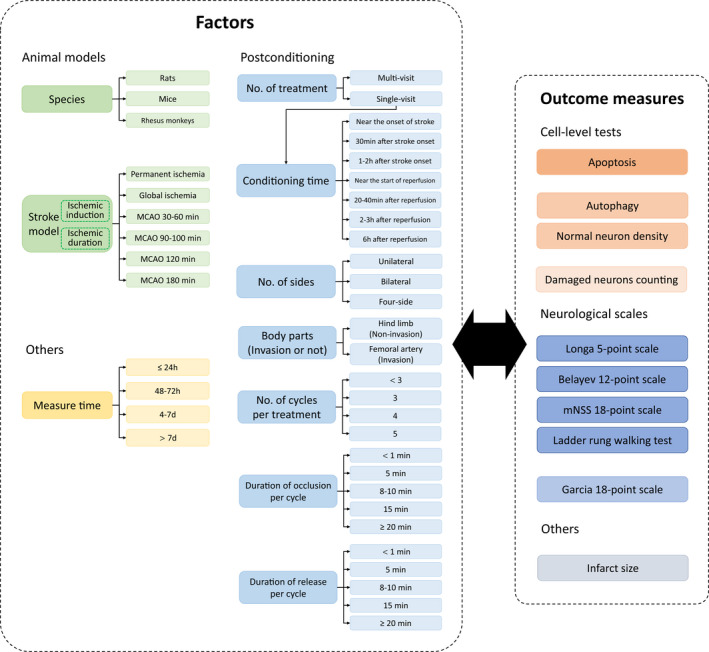
Summary of factors and outcome measures. The factors include the categories of animal model (green), postconditioning (sky blue), and measure time (yellow). Notably, measure time actually cannot influence the real treatment effect but likely affects the outcome measures, so we included it and recorded the measure time points with the stroke onset as a reference; the factor of conditioning time is only in the subgroup of single‐visit treatment because multi‐visit treatments have multiple times of conditioning, which are difficult to count and of little significance. Outcome measures contain cell‐level tests (orange), neurological scales (dark blue), and infarct size (gray). For the cell‐level tests and neurological scales have different methods with different principles, they would be merged into three groups and two groups based on the criterion that there is no statistical difference within groups but significant differences between groups (distinguished by the shades of color), respectively. d, days; h, hours; No., numbers; min, minutes.

The identified studies were screened and selected based on specific criteria through different phases in line with the Preferred Reporting Items for Systematic Reviews and Meta‐Analyses (PRISRMA) Statement^12^ (Figure 1). Firstly, the records from different sources were integrated and duplicates were removed, then those unrelated to the topic were excluded by screening their abstracts and titles. Next, the full‐text articles of all candidate records were required as much as possible, which were further assessed for eligibility, and the abstract‐only records were also excluded. The eligibility criteria are summarized as follows:

1. Article type: research article, not literature review;
2. Study subject: experimental model of animal, not human being;
3. Study design: randomized study with treatment and control groups;
4. Disease model: acute ischemic stroke model, not chronic ischemia, hemorrhage, or other brain injury;
5. Treatment: including the trials only administered by RIPostC, not in combination with others, and RIPostC conducted in the limbs instead of others such as gastric artery;
6. Outcome measures (Figure 2): containing at least one of the measures of neurological scales, infarct size, and indicators of cell level including autophagy (terminal deoxynucleotidyl transferase dUTP nick end labeling, TUNEL), apoptosis (microtubule‐associated protein 1A light‐chain 3‐II level, LC3‐II; LC3‐II/LC3‐I ratio), normal‐neuron density (thionine staining and immunohistochemistry for neuronal nuclei, NeuN; Nissl staining), and damaged‐neuron counting (hematoxylin and eosin staining, H&E);
7. Data: having corresponding data or figures of the above categories and data consistency in the article;
8. Language: written in English or Chinese.

### 2.3 Quality assessment

The risk of bias in each included study was assessed using the revised version of the Cochrane risk‐of‐bias tool for randomized trials (RoB 2).^13^ Besides, extra publication bias was analyzed by the Egger's test^14^ and Begg's rank test^15^ in MedCalc 20.^16^ The domains of bias in the RoB 2 tool were: (1) bias arising from the randomization process; (2) bias due to deviations from intended interventions; (3) bias due to missing outcome data; (4) bias in the measurement of the outcome; (5) bias in the selection of the reported result; (6) overall bias, and each domain was assigned one of three levels according to the criteria: (a) low risk; (b) some concerns; (c) high risk.

Study quality was also assessed using the published criteria^17^ for stroke animal experiments recommended by the research group of the Collaborative Approach to Meta Analysis and Review of Animal Experimental Studies (CAMARADES, https://www.ed.ac.uk/clinical‐brain‐sciences/research/camarades). The checklist comprised: (1) peer‐reviewed publication; (2) statement of control of temperature; (3) random allocation to treatment or control; (4) blinded induction of ischemia; (5) blinded assessment of outcome; (6) use of anesthetic without significant intrinsic neuroprotective activity; (7) appropriate animal model (transient, permanent, embolic or photothrombotic models); (8) sample size calculation; (9) compliance with animal welfare regulations; (10) statement of potential conflict of interests. Each above item was assigned one point, and thus each study was given a score out of a total 10 points.

### 2.4 Data extraction

From each included study, the data independently extracted by four investigators (half by Z.W. and J.T., half by Z.C. and Y.C.) are as follows (Figure 2).

1. Basic information of study article: country, published year, used language;
2. Preparation for animal model: species, method of ischemic induction, duration of ischemia;
3. Parameters of postconditioning: conditioning time, number of treatment (single‐visit or multi‐visit), number of sides (unilateral, bilateral, or four‐side), body part (hind limb or femoral artery, indicating noninvasion or invasion), number of cycles per treatment, duration of occlusion per cycle, duration of release per cycle; and measure time (stroke onset as the start of timing);
4. Data related to outcome measures: method with the unit of calculation, time of measurement, the number of animals, mean and standard deviations (SD) of measure in control and treatment groups.

If the requested data were only presented by graphs, the exact values would be measured using the digital ruler in PDF viewer and further determined by the average from different investigators. Notably, the “postconditioning” in our study was generalized, consisting of narrow “postconditioning” starting after reperfusion and “perconditioning” starting between the onset of occlusion and reperfusion. For the convenience of statistics and analysis, one record of the experiment is defined as one from a unique study with the only ischemic model, RIPostC, and measures. In other words, the above‐mentioned related data and parameters cannot be completely consistent between different experiments. The neurological scales used in the included studies were many and various, but we only counted those with a high frequency of use and clear, precise scoring criteria (Document S1).

### 2.5 Data analysis

#### 2.5.1 Meta‐analysis methods

The meta‐analysis was performed in the software of Cochrane Review Manager 5.4.1 (RevMan 5.4.1).^18^ Due to the continuous attribute and different measurement scales of outcome measures, the treatment effect in the study was presented by standardized mean difference (SMD) with 95% confidence intervals. The statistical algorithms used in our study including SMD (Hedges' adjusted G method^19^), weights to each experiment (inverse‐variance method, IV), heterogeneity (*Q* test^20^ and *I* ^2^ method^21^), random‐effect model,^22^ test for the presence of overall effect (*Z* test), and test for comparison of intragroup (*Q* test^20^ and *I* ^2^ method^21^) were by default in RevMan 5.4.1. Statistically significant difference was considered as *p* < 0.05.

#### 2.5.2 Data preprocessing and meta‐analysis strategy

Given the complexity and diversity of the extracted data, they were preprocessed before meta‐analysis, including normalization and grouping (see Document S2 with Figure S1 for details). After preprocessing and grouping (Figure 2), there are still scarce experimental records of certain groups under specific factors. Inspired by the idea of sensitivity analysis based on the one‐by‐one elimination method, the records that cause the above situations and unstable results would be appropriately eliminated during the analysis. Throughout the analysis, any particular process will be specifically pointed out later.

## 3 RESULTS

### 3.1 Study characteristics

A total of 1738 records were identified from those mentioned search engines and other sources, of which 57 studies (Table S1) were assessed for full‐text eligibility and included in further meta‐analysis. The included studies were carried out in nine countries (China, number of studies[*N*] = 44; USA, *N* = 4; Canada, *N* = 3; Italy, *N* = 2; India *N* = 1; Japan, *N* = 1; Qatar *N* = 1; Slovakia, *N* = 1), all published after the year of 2009 (2009–2012, *N* = 10; 2013–2016, *N* = 28; 2017–2020, *N* = 19) written by English (*N* = 47) or Chinese language (*N* = 10), and comprised of 224 valid experiment records.

Among all of included experiments, outcome measure of 91 experiments was infarct size (2,3,5‐triphenyltetrazolium chloride staining (TTC), number of experimental records (*n*) = 79; H&E, *n* = 1; Cresyl violet staining (CV), *n* = 1; T2 weighted magnetic resonance imaging (T2‐MRI), *n* = 3; diffusion weighted imaging (DWI), *n* = 6; phase‐contrast microscopy, *n* = 1); 92 experiments were measured by neurological scales including Longa 5‐point scale (*n* = 19), modified neurological severity scores (mNSS) 18‐point scale (*n* = 25), Belayev 12‐point scale (*n* = 10), Ladder rung walking test (*n* = 3), and Garcia 18‐point scale (*n* = 35).

The remaining 41 experiments were assessed by four cell‐level tests, none of which showed statistically significant differences in sampling regions, including apoptosis (TUNEL in hippocampal cornu ammonis [CA1] regions, *n* = 10; TUNEL in penumbra regions, *n* = 11; *p* = 0.82), autophagy (LC3‐II or LC3‐II/LC3‐I in ischemia regions, *n* = 2; or in penumbra regions, *n* = 7; *p* = 0.51), normal‐neuron density (Nissl staining and NeuN in CA1 regions, *n* = 3; Nissl staining and NeuN staining in ischemia/penumbra regions, *n* = 6; *p* = 0.15) and damaged‐neuron counting (H&E in CA1 region, *n* = 1; H&E in ischemia region, *n* = 1; *p* = 0.27).

### 3.2 Outcome measures

The treatment effect expressed by SMD based on the outcome measure of infarct size (Table 1, Figure S2) is 3.32 (95% confidence interval (CI): [2.86, 3.78]). There are three species including rats, mice, and rhesus monkeys in the data on infarct size. Given that species and experimental procedures used in the study of rhesus monkeys^23^ were quite different from those using rodents, and the former did not show therapeutic efficacy (SMD: −0.22 [−0.81, 0.37], *p* = 0.46), our study tried not to include rhesus monkey‐related data and the effect size rises to 3.60 (95% CI: [3.12, 4.07]).

**TABLE 1:** Treatment effects of different factors based on the outcome measure of infarct size

Overall effect/factors	No. of experimental records	No. of animals in treatment group	No. of animals in control group	Treatment effect (SMD [95% CI])	*p*
Overall effect	91	730	720	3.32 [2.86, 3.78]***	
Overall effect (without Rhesus monkeys)	85	700	702	3.60 [3.12, 4.07]***	
Species					
Rats	72	592	577	3.89 [3.35, 4.44]***	<0.00001***
Mice	13	108	125	2.22 [1.41, 3.03]***	
Rhesus monkeys	6	30	18	−0.22 [−0.81, 0.37]	
Stroke models					
Permanent ischemia	10	92	100	4.38 [2.82, 5.93]***	0.59
MCAO 30–60 min	15	156	172	3.30 [2.13, 4.46]***	
MCAO 90–100 min	39	298	275	3.65 [2.93, 4.38]***	
MCAO 120 min	21	154	155	3.26 [2.50, 4.02]***	
No. of treatments					
Multi‐visit	5	51	47	2.56 [1.06, 4.07]***	0.17
Single‐visit	80	649	656	3.68 [3.18, 4.18]***	
Conditioning time					
Near the onset of stroke	10	88	87	2.65 [1.74, 3.56]***	0.10
30 min after stroke onset	1	8	8	6.70 [3.85, 9.55]***	
1–2 h after stroke onset	8	82	83	3.49 [2.10, 4.89]***	
Near the start of reperfusion	47	369	356	3.83 [3.11, 4.56]***	
20–40 min after reperfusion	5	34	31	3.50 [1.19, 5.82]**	
2–3 h after reperfusion	5	38	60	4.36 [1.98, 6.74]***	
6 h after reperfusion	4	30	30	3.68 [3.18, 4.18]***	
No. of sides					
Unilateral	24	200	223	3.42 [2.57, 4.26]***	0.62
Bilateral	61	500	479	3.68 [3.10, 4.25]***	
Body parts (invasion or not)					
Hind limb (noninvasion)	32	302	316	3.52 [2.76, 4.28]***	0.79
Femoral artery (invasion)	53	398	386	3.66 [3.04, 4.27]***	
No. of cycles per treatment					
<3	15	94	80	3.69 [2.43, 4.96]***	<0.00001***
3	61	531	526	3.89 [3.32, 4.47]***	
4	5	44	45	2.55 [0.58, 4.52]***	
5	4	31	51	1.38 [0.66, 2.10]***	
Duration of occlusion per cycle					
<1 min	3	22	21	1.65 [−0.12, 3.42]	0.0007***
5 min	22	181	194	2.43 [1.68, 3.19]***	
8–10 min	46	396	385	4.04 [3.37, 4.71]***	
15 min	7	59	67	4.64 [2.52, 6.77]***	
≥20 min	7	42	35	5.75 [3.59, 7.91]***	
Duration of release per cycle					
<1 min	3	22	21	1.65 [−0.12, 3.42]	0.003**
5 min	22	181	194	2.43 [1.68, 3.19]***	
8–10 min	43	378	379	3.97 [3.28, 4.66]***	
15 min	7	59	67	4.64 [2.52, 6.77]***	
≥20 min	1	6	5	5.91 [2.61, 9.22]***	
Measure time					
≤24 h	57	488	454	3.33 [2.77, 3.89]***	0.04*
48–72 h	23	174	199	4.39 [3.32, 5.46]***	
4–7 days	3	48	47	2.12 [0.86, 3.38]**	
>7 days	2	20	20	6.34 [1.12, 11.55]*	

*Note*: There are great differences in species between rhesus monkeys and rodents, with different experimental designs and methods of ischemic induction (rhesus monkeys: MCAO 180 min and remote postconditioning of four‐size limbs). Besides, the results related to rhesus monkeys show no significant and effective treatment effect; therefore, except for the factor of species, the other factors do not include the experimental data of rhesus monkeys in the analysis. The column of *p*, statistics for testing subgroup differences within one factor; *p < 0.05; **p < 0.01; ***p < 0.001.

Abbreviations: CI, confidence interval; d, days; h, hours; MCAO, middle cerebral artery occlusion; min, minutes; No., numbers; SMD, standardized mean difference.

For Garcia 18‐point scale and cell‐level tests of autophagy and normal‐neuron density, smaller neurological damages correspond to larger values, which were preprocessed before the following meta‐analysis in our study. According to the criterion that there is no statistical difference within groups but significant differences between groups, all kinds of included scales were finally divided into two groups (Table 2): Group A (Figure S3; SMD: 1.58 [1.27, 1.89]) including Longa 5‐point scale,^24^ Belayev 12‐point scale,^25^ mNSS 18‐point scale^26^ and Ladder rung walking test^27^, ^28^ (*p* = 0.23, intragroup test), and Group B (Figure S4) only with Garcia 18‐point scale^29^ (SMD: 3.86 [3.18, 4.55]), with *p* < 0.00001 between two groups. All cell tests could be divided into three groups (Table 3): Group C (Figure S5) only with damaged‐neurons counting (SMD: 8.42 [4.99, 11.86]), Group D (Figure S6) only with autophagy (SMD: 2.13 [0.55, 3.71]), and Group E (Figure S7; SMD: 3.82 [2.93, 4.70]) including apoptosis and normal‐neuron density (*p* = 0.16, intragroup test), with *p* = 0.004 among three groups.

**TABLE 2:** Treatment effects of different factors based on the outcome measure of neurological scales

Overall effect/factors	No. of experimental records	No. of animals in treatment group	No. of animals in control group	Treatment effect (SMD [95% CI])	*p*
Group A					
Overall effect	57	532	506	1.58 [1.27, 1.89]***	0.23
Longa 5‐point scale	19	205	198	1.79 [1.21, 2.38]***	
Belayev 12‐point scale	10	105	104	0.93 [0.21, 1.65]*	
mNSS 18‐point scale	25	194	176	1.74 [1.31, 2.17]***	
Ladder rung walking test	3	28	28	1.22 [−0.31, 2.74]	
Species					
Rats	51	483	456	1.54 [1.22, 1.85]***	0.55
Mice	6	65	66	1.93 [0.70, 3.17]**	
Stroke models					
Permanent ischemia	5	54	54	2.25 [1.02, 3.48]***	0.69
MCAO 30–60 min	3	26	26	1.58 [−0.55, 3.72]	
MCAO 90–100 min	32	345	319	1.49 [1.13, 1.84]***	
MCAO 120 min	16	107	107	1.69 [0.94, 2.44]***	
No. of treatments					
Multi‐visit	6	38	38	3.19 [2.41, 3.96]***	<0.0001***
Single‐visit	51	494	468	1.42 [1.11, 1.73]***	
Conditioning time					
Near the onset of stroke	10	108	108	0.88 [0.44, 1.32]***	<0.00001***
30 min after stroke onset	1	15	15	1.19 [0.41, 1.98]**	
1–2 h after stroke onset	12	131	114	1.35 [0.97, 1.73]***	
Near the start of reperfusion	27	232	223	1.90 [1.36, 2.43]***	
20–40 min after reperfusion	1	8	8	−2.73 [−4.19, −1.26]***	
No. of sides					
Unilateral	22	252	227	1.39 [1.00, 1.77]***	0.22
Bilateral	35	280	279	1.76 [1.31, 2.21]***	
Body parts (invasion or not)					
Hind limb (noninvasion)	32	270	263	1.79 [1.31, 2.26]***	0.25
Femoral artery (invasion)	25	262	243	1.42 [1.02, 1.82]***	
No. of cycles per treatment					
<3	2	44	44	2.04 [0.19, 3.89]*	0.68
3	50	445	418	1.54 [1.21, 1.87]***	
4	3	26	26	1.13 [−0.41, 2.67]	
5	2	17	18	3.01 [0.06, 5.95]	
Duration of occlusion per cycle					
5 min	22	245	220	1.18 [0.81, 1.55]***	0.01*
8–10 min	34	275	274	2.02 [1.52, 2.52]***	
15 min	1	12	12	0.79 [−0.04, 1.63]	
Duration of release per cycle					
5 min	22	245	220	1.18 [0.81, 1.55]***	0.01*
8–10 min	34	275	274	2.02 [1.52, 2.52]***	
15 min	1	12	12	0.79 [−0.04, 1.63]	
Measure time					
≤24 h	27	271	260	1.11 [0.69, 1.53]***	0.005**
48–72 h	11	88	85	1.79 [1.10, 2.49]***	
4–7 days	9	89	81	1.92 [1.19, 2.66]***	
>7 days	9	69	65	3.03 [1.93, 4.13]***	
Group B					
Overall effect	35	304	304	3.86 [3.18, 4.55]***	
Garcia 18‐point scale	35	304	304	3.86 [3.18, 4.55]***	0.0002***
Sun et al., 2012	18	144	144	5.42 [4.15, 6.69]***	
Studies without Sun et al., 2012	17	160	160	2.68 [1.99, 3.38]***	
Species					
Rats	35	304	304	3.86 [3.18, 4.55]***	
No. of treatments					
Single‐visit	35	304	304	3.86 [3.18, 4.55]***	
No. of sides					
Bilateral	35	304	304	3.86 [3.18, 4.55]***	
Stroke models					
Permanent ischemia	1	6	6	3.10 [1.21, 5.00]**	0.004** (0.84)
MCAO 30–60 min	7	90	90	2.87 [3.97, 1.76]***	
MCAO 90–100 min	18 (0)	144 (0)	144 (0)	5.42 [4.15, 6.69]*** (/)	
MCAO 120 min	9	64	64	2.53 [1.50, 3.56]***	
Conditioning time					
Near the start of reperfusion	16	152	152	2.84 [2.17, 3.52]***	<0.00001*** (<0.0001***)
20–40 min after reperfusion	1	8	8	0.20 [−0.79, 1.18]	
2–3 h after reperfusion	9 (0)	72 (0)	72 (0)	4.85 [3.04, 6.67]*** (/)	
6 h after reperfusion	9 (0)	72 (0)	72 (0)	5.88 [4.28, 7.49]*** (/)	
Body parts (invasion or not)					
Hind limb (noninvasion)	13	126	126	2.66 [1.95, 3.37]***	0.0006*** (0.82)
Femoral artery (invasion)	22 (4)	178 (34)	178 (34)	4.91 [3.79, 6.03]*** (2.92 [0.70, 5.14]*)	
No. of cycles per treatment					
3	31 (13)	280 (136)	280 (136)	4.07 [3.30, 4.84]*** (2.71 [1.87, 3.55]***)	0.01** (0.86)
4	4	24	24	2.60 1.74, 3.47]***	
Duration of occlusion per cycle					
<1 min	6 (0)	48 (0)	48 (0)	2.93 [1.62, 4.25]*** (/)	<0.0001*** (/)
5 min	6 (0)	48 (0)	48 (0)	8.86 [6.57, 11.15]*** (/)	
8–10 min	23 (17)	208 (160)	208 (160)	3.27 [2.56, 3.98]*** (2.68 [1.99, 3.38]***)	
Duration of release per cycle					
<1 min	6 (0)	48 (0)	48 (0)	2.93 [1.62, 4.25]*** (/)	<0.0001*** (/)
5 min	6 (0)	48 (0)	48 (0)	8.86 [6.57, 11.15]*** (/)	
8–10 min	23 (17)	208 160)	208 (160)	3.27 [2.56, 3.98]*** (2.68 [1.99, 3.38]***)	
Measure time					
≤24 h	14 (8)	125 (77)	125 (77)	3.34 [2.28, 4.41]*** (2.76 [1.53, 4.00]***)	0.04* (0.99)
48–72 h	19 (7)	156 (60)	156 (60)	4.55 [3.50, 5.61]*** (2.69 [1.66, 3.72]***)	
4–7 days	2	23	23	2.80 [1.95, 3.66]***	

*Note*: All kinds of neurological scales can be divided into two groups: Group A including Longa 5‐point scale, Belayev 12‐point scale, mNSS 18‐point scale, and Ladder rung walking test (*p* = 0.23, intragroup test) and Group B only with Garcia 18‐point scale, while *p* < 0.00001 between two groups. In Group B (Garcia 18‐point scale), the data from study of Sun et al., 2012 (SMD: 5.42 [4.15, 6.69]) have a quite larger effect size than others (SMD: 2.68 [1.99, 2.68]) with a very significant difference (*p* = 0.0002), so for the factors with more than one treatment the table, the table not only illustrates the statistics of all data in the group, but also those excluding study of Sun et al.,^30^ (values of each factor in parentheses ), “0” or “/” means none. The column of *p*, statistics for testing subgroup differences within one factor; *p < 0.05; **p < 0.01; ***p < 0.001.

Abbreviations: CI, confidence interval; d, days; h, hours; MCAO, middle cerebral artery occlusion; min, minutes; No., numbers; SMD, standardized mean difference.

**TABLE 3:** Treatment effects of different factors based on the outcome measure of cell‐level tests

Overall effect/factors	No. of experimental records	No. of animals in treatment group	No. of animals in control group	Treatment effect (SMD [95% CI])	*p*
Group C					
Overall effect	2	13	13	8.42 [4.99, 11.86]***	
Damaged‐neuron counting	2	13	13	8.42 [4.99, 11.86]***	
Group D					
Overall effect	9	45	45	2.13 [0.55, 3.71]**	
Autophagy	9	45	45	2.13 [0.55, 3.71]**	<0.00001***
Chen et al., 2018	1	6	6	−3.54 [−5.61, −1.47]***	
Studies without Chen et al., 2018	8	39	39	2.67 [1.64, 3.70]***	
Species					
Rats	8 (7)	39 (33)	39 (33)	1.94 [0.22, 3.65]*(2.53 [1.41, 3.64]***)	0.21 (0.35)
Mice	1	6	6	3.68 [1.56, 5.81]***	
Stroke models					
MCAO 90–100 min	7 (6)	34 (28)	34 (28)	1.97 [−0.10, 4.03] (2.75 [1.33, 4.18]***)	0.61 (0.88)
MCAO 120 min	2	11	11	2.60 [1.31, 3.89]***	
No. of treatments					
Single‐visit	9 (8)	45 (39)	45 (39)	2.13 [0.55, 3.71]** (2.67 [1.64, 3.70]***)	
Conditioning time					
30 min after stroke onset	1	4	4	3.79 [0.84, 6.74]*	0.29 (0.45)
Near the start of reperfusion	8 (7)	41 (35)	41 (35)	1.96 [0.27, 3.65]* (2.58 [1.47, 3.68]***)	
No. of sides					
Unilateral	1 (0)	6 (0)	6 (0)	−3.54 [−5.61, −1.47]*** (/)	<0.00001*** (/)
Bilateral	8	39	39	2.67 [1.64, 3.70]***	
Body parts (invasion or not)					
Hind limb (noninvasion)	3 (2)	16 (10)	16 (10)	0.26 [−3.45, 3.98] (2.07 [−1.20, 5.35])	0.19 (0.66)
Femoral artery (invasion)	6	29	29	2.83 [1.96, 3.71]***	
No. of cycles per treatment					
3	7 (6)	35 (29)	35 (29)	2.17 [0.15, 4.19]* (2.83 [1.96, 3.71]***)	0.96 (0.66)
4	2	10	10	2.07 [−1.20, 5.35]	
Duration of occlusion per cycle					
8–10 min	8	39	39	2.67 [1.64, 3.70]***	<0.00001*** (/)
15 min	1 (0)	6 (0)	6 (0)	−3.54 [−5.61, −1.47]*** (/)	
Duration of release per cycle					
8–10 min	8	39	39	2.67 [1.64, 3.70]***	<0.00001*** (/)
15 min	1 (0)	6 (0)	6 (0)	−3.54 [−5.61, −1.47]*** (/)	
Measure time					
≤24 h	9 (8)	45 (39)	45 (39)	2.13 [0.55, 3.71]** (2.67 [1.64, 3.70]***)	
Group E					
Overall effect	30	243	225	3.70 [2.82, 4.59]***	0.29
Apoptosis	21	122	122	3.22 [2.63, 3.82]***	
Normal‐neuron density	9	121	103	4.33 [2.36, 6.30]***	
Species					
Rats	27	165	165	3.79 [3.07, 4.50]***	0.12
Mice	3	84	66	1.65 [−0.93, 4.24]	
Stroke models					
Global ischemia	6	40	40	4.16 [2.90, 5.41]***	0.27
MCAO 30–60 min	4	86	68	1.90 [−0.29, 4.10]	
MCAO 90–100 min	6	38	38	4.66 [2.26, 7.07]***	
MCAO 120 min	14	79	79	3.46 [2.57, 4.35]***	
No. of treatments					
Multi‐visit	9	118	100	3.28 [1.59, 4.98]***	0.54
Single‐visit	22	131	131	3.87 [3.06, 4.68]***	
Conditioning time					
Near the onset of stroke	1	8	8	2.73 [1.26, 4.19]***	0.01*
Near the start of reperfusion	18	107	107	3.96 [3.07, 4.85]***	
20–40 min after reperfusion	1	5	5	1.27 [−0.16, 2.70]	
6 h after reperfusion	1	5	5	4.90 [1.88, 7.93]**	
No. of sides					
Unilateral	4	25	25	5.27 [1.56, 8.98]**	0.38
Bilateral	26	218	200	3.58 [2.65, 4.50]***	
Body parts (invasion or not)					
Hind limb (noninvasion)	15	154	136	3.42 [2.15, 4.69]***	0.59
Femoral artery (invasion)	15	89	89	3.85 [2.92, 4.79]***	
No. of cycles per treatment					
3	27	225	207	3.48 [2.58, 4.38]***	0.04*
4	3	18	18	5.81 [3.80, 7.82]***	
Duration of occlusion per cycle					
5 min	3	15	15	2.22 [0.66, 3.79]**	0.20
8–10 min	20	180	162	3.81 [2.65, 4.97]***	
15 min	7	48	48	3.86 [2.78, 4.94]***	
Duration of release per cycle					
5 min	3	15	15	2.22 [0.66, 3.79]**	0.20
8–10 min	20	180	162	3.81 [2.65, 4.97]***	
15 min	7	48	48	3.86 [2.78, 4.94]***	
Measure time					
≤24 h	17	98	98	3.35 [2.54, 4.15]***	0.16
48–72 h	4	23	23	3.19 [2.10, 4.28]***	
4–7 days	3	22	22	5.52 [3.70, 7.35]***	
>7 days	6	100	82	3.35 [1.18, 5.52]**	

*Note*: All kinds of cell‐level tests can be divided into three groups: Group C including damaged‐neuron counting, Group D including autophagy, and Group E including apoptosis and normal‐neuron density (*p* = 0.29, intragroup test), while *p* = 0.004 among three groups. Group C (damaged‐neurons counting) just has two experimental records, so only the overall effect is calculated (SMD: 8.42 [4.99, 11.86]), and each factor is no longer analyzed separately. In Group D (autophagy), the treatment effect of study of Chen et al.,2018^31^ (SMD: −3.54 [−5.61, −1.47]) is exactly the opposite to those of other studies (SMD: 2.67 [1.64, 3.70]), with a significant statistical difference (*p* < 0.00001); therefore, the table not only presents the statistics of all data in the group, but also those excluding study of Chen et al., 2018 (values of each factor in parentheses); “0” or “/” means none. The column of *p*, statistics for testing subgroup differences within one factor; *p < 0.05; **p < 0.01; ***p < 0.001.

Abbreviations: CI, confidence interval; d, days; h, hours; MCAO, middle cerebral artery occlusion; min, minutes; No., numbers; SMD, standardized mean difference.

Just from the point of view of overall effect sizes, the treatment effect measured by Garcia 18‐point scale is generally higher than that of other scales. In the data on cell‐level tests, the treatment effect represented by damaged‐neurons counting is the largest, followed by apoptosis and normal‐neuron density, and the worst was autophagy. On the other hand, we noticed a strikingly significant difference (*p* = 0.0002) between the outcomes of the study of Sun et al.,^30^ (SMD: 5.42 [4.15, 6.69]) and the rest of the studies (SMD: 2.68 [1.99, 2.68]) in Group B (Garcia 18‐point scale), so data of Group B with or without Sun et al., 2012 were both analyzed and presented in Table 2. Even in Group B excluding data from Sun et al., 2012, there remained a statistical difference between Group A and B (*p* = 0.004). Due to just two experimental records in Group C (damaged‐neurons counting), only the overall effect was calculated and each specific factor was no longer analyzed separately. In Group D (autophagy), the treatment effect of the study by Chen et al.,^31^ (SMD: −3.54 [−5.61, −1.47]) was exactly the opposite to those of other studies (SMD: 2.67 [1.64, 3.70]) with a significant difference (*p* <0.00001).

### 3.3 Animal models

For species, the treatment effect only measured by infarct size shows a statistical difference between rats and mice (*p* = 0.0008) among three kinds of outcome measures, and the effect size is higher in mice (SMD: 3.89 [3.35, 4.44]) than in rats (SMD: 2.22 [1.41, 3.03]). While there was no difference between various varieties within the same species (rats with the varieties of Sprague–Dawley (SD) rats and Wistar rats, *p* = 0.96; mice with the varieties of C57BL/6 mice and CD mice, *p* = 0.11). Interestingly, stroke models determined by ischemic induction and duration do not show statistical differences in any of the measures (Tables 1, 2, 3).

### 3.4 Measure time

Measure time shows a difference in effects based on infarct sizes (*p* = 0.04) and Group A in neurological scales (*p* = 0.005), does not in cell‐level tests (*p* = 0.16 in Group E). The effect based on infarct sizes in measure time between 48 and 72 h is almost most obvious (SMD: 4.39 [3.32, 5.46]) then declines, and the data in Table 1 show that effect size reaches the maximal measured after 7 days of ischemia onset, nevertheless, the reliability of which is limited because of only two records with measure time of after‐seven‐day (Table 1). But the effect size is greater when the time point measured based on Group A in neurological scales from the onset of the stroke is longer (Table 2).

### 3.5 Postconditioning

Among postconditioning‐related factors, there is no significant difference in the number of sides and body parts (Tables 1, 2, 3). For the number of treatments, the results of Group A in neurological scale show that multi‐visit (SMD: 3.19 [2.41, 3.96]) is better than single‐visit (SMD: 1.42 [1.11, 1.73]) with significance (*p* <0.0001), but other measures do not have significant differences and the effect size of single‐visit (infarct size, SMD: 3.68 [3.18, 4.18]; Group E in cell‐level tests: 3.87 [3.06, 4.68]) was generally higher than multi‐visit (infarct size, SMD: 2.56 [1.06, 4.07]; Group E in cell‐level tests, SMD: 3.28 [1.59, 4.98]).

**FIGURE 3:**
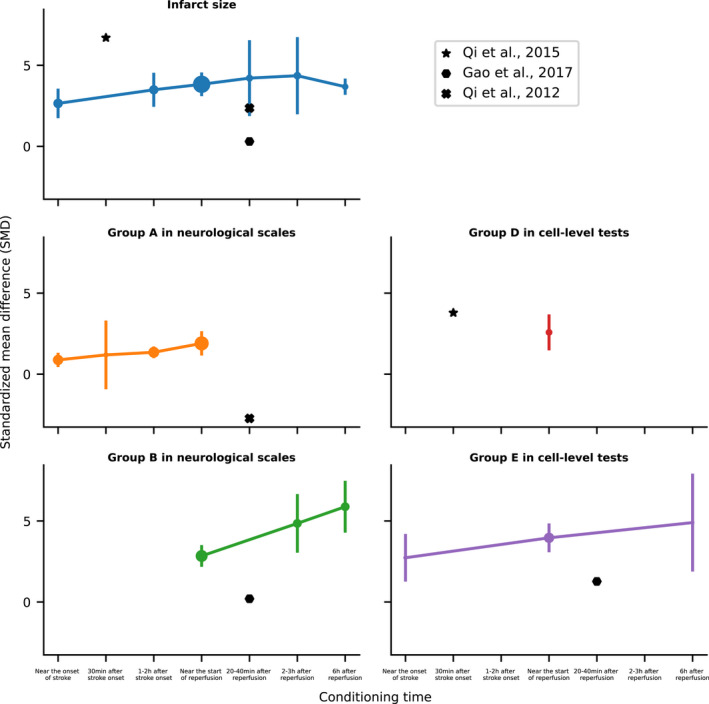
Treatment effects for the factor of conditioning time based on different outcome measures. Treatment effects presented by standardized mean difference (SMD in *Y*‐axis) along with different conditioning times (*X*‐axis) are represented by lines with error bars (bars: 95% confidence intervals; solid points: number of animals, the larger the point is, the more number is). And the outcome measures include infarct size (upper left, blue; *p* = 0.32, intragroup test); Group A in neurological scales with Longa 5‐point scale,^24^ Belayev 12‐point scale,^25^ mNSS 18‐point scale,^26^ and Ladder rung walking test^27^, ^28^ (middle left, orange; *p* = 0.02); Group B in neurological scales with Garcia 18‐point scale^29^ (bottom left, green; *p* = 0.0008); Group D in cell‐level tests with autophagy level (middle right, red); and Group E in cell‐level tests with apoptosis and normal‐neuron density (bottom right, purple; *p* = 0.27), all of which almost follow the trend that the treatment effect is better when the time point of conditioning is longer from the onset of stroke. But some of studies were not subject to the trend and drawn out particularly as outliers including Qi et al.,^34^ (star points); Gao et al.,^35^ (hexagon points); Qi et al.,^36^ (“X” points). d, days; h, hours; min, minutes.

The following parameters have significant impacts on the treatment effect of postconditioning including conditioning time, number of cycles per treatment, duration of occlusion, and release per cycle. Firstly, for conditioning time (Figure 3), most of the included experiments were selected near the onset of stroke (*n* = 21), 1–2 h after stroke onset (*n* = 20), and near the start of reperfusion (*n* = 117). Several studies have performed postconditioning experiments at time points not less than 2 h after reperfusion (Yang et al.,^32^ *n* = 1; Ren et al.,^33^ *n* = 2; Sun et al.,^30^ *n* = 25). Based on the relevant data at the aforementioned time points, the outcome measures of infarct size (*p* = 0.32) and Group E of cell‐level tests (*p* = 0.27) did not show statistical differences among different treatment levels, but Group A (*p* = 0.04) and Group B (*p* = 0.0008) of neurological scales did show significance. Besides, a small number of studies would select less frequently used conditioning times, such as 30 min after stroke onset (number of studies, *N* = 3; number of experiments, *n* = 3) or 20–40 min after reperfusion onset (*N* = 3, *n* = 8), among which some studies (black solid points in Figure 3: Qi et al.,^34^; Gao et al.^35^; Qi et al.,^36^) performed as outliers. To be specific, postconditioning 30 min after stroke onset presented by autophagy level in the study of Qi et al.,^34^ (SMD: 3.79 [0.84, 6.74]) worked better than other studies near the start of reperfusion (SMD: 2.58 [1.47, 3.68]) in Group D; the treatment effects of postconditioning 30 min after reperfusion in the studies of Gao et al., and Qi et al., 2012 were worse than those near the start of reperfusion,^35^, ^36^ and the Belayev 12‐point scale score in the treatment group was even a bit higher than the control group in Qi et al.,^36^, which means such conditioning had no treatment effect (SMD: ‐2.73 [‐4.19, ‐1.26]). Adding these outliers' data into the intragroup test of the factor, Group E was transformed into a statistical difference (*p* = 0.01, Table 3).

**FIGURE 4:**
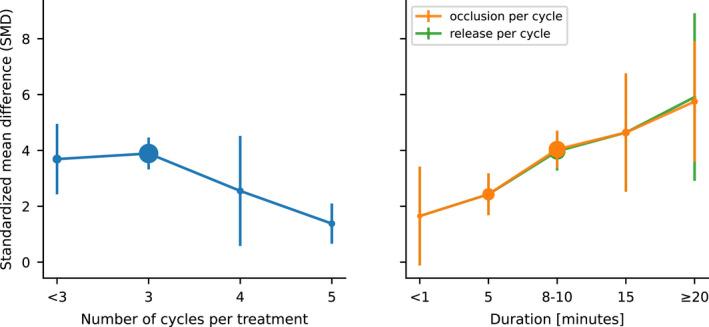
Treatment effects based on infarct size for factors including number of cycles per treatment, duration of occlusion, and release per cycle. Treatment effects presented by standardized mean difference (SMD in *Y*‐axis) along with different treatment levels of factors including number of cycles per treatment (left, blue; *p* < 0.00001, intragroup test), duration of occlusion (*p* = 0.0007), and release per cycle (right, orange; *p* = 0.003) are represented by lines with error bars (bars: 95% confidence intervals; solid points: number of animals, the larger the point is, the more number is). As we can see, three cycles per treatment work best among all possible treatment levels in the factor, while effect sizes are generally positive correlation with the duration of occlusion and release per cycle.

For other significant postconditioning‐related parameters, the data based on infarct size are larger and have more treatment levels than other measures. According to Table 1, the number of cycles per treatment (*p* < 0.00001), duration of occlusion (*p* = 0.0007), and release (*p* = 0.003) per cycle all have differences in statistics; performing three times works best for the number of cycles per treatment (SMD: 3.89 [3.32, 4.47]), while effect sizes are generally positive correlation with the duration of occlusion and release per cycle (Figure 4). On the other hand, the data based on neurological scales and cell‐level tests are mainly focused on a few treatment levels (Table 2–3). There are 3 (*n* = 117) or 4 (*n* = 12) cycles per treatment with little statistical difference in effect between them (except Group E in cell‐level tests, *p* = 0.04). The duration of occlusion and release per cycle is 5 min (*n* = 31) or 8–10 min (*n* = 85), with a few being 15 min (*n* = 9) or less than 1 min (*n* = 6). From the results, 8–10 min (Group A in neurological scales, SMD: 2.02 [1.52, 2.52]; Group E in cell‐level tests: 3.81 [2.65, 4.97]) is better than 5 min (Group A in neurological scales, SMD: 1.18 [0.81, 1.55]; Group E in cell‐level tests: 2.22 [0.66, 3.79]).

### 3.6 Study quality and risk of bias

In all of 57 include studies, the overall risk of bias (Table S2) assessed with the RoB 2 tool was 51 (89.5%) of low risk, 4 (7.0%) of Some concerns and 2 (3.5%) of high risk (Table S2). However, the publication bias based on various outcome measures show significant (Egger's test: infarct size, *p* < 0.0001; Group A in neurological scales, *p* < 0.0001; Group B, *p* < 0.0001; Group C in cell‐level tests, *p* < 0.0001; Group D, *p* = 0.25; Group E, *p* < 0.0001. Begg's test: infarct size, *p* < 0.0001; Group A in neurological scales, *p* < 0.0001; Group B, *p* < 0.0001; Group C in cell‐level tests, *p* = 0.32; Group D, *p* = 0.037; Group E, *p* < 0.0001). Furthermore, the mean of study quality score calculated using CAMARADES (Table S3) is 7.47 with SD of 1.40, the distribution of which is 5–6 (*N* = 15), 7–8 (*N* = 26), 9–10 (*N* = 16). It should be additionally pointed out that only two studies^37^, ^38^ received a full score.

## 4 DISCUSSION

Based on our systematic meta‐analysis, remote ischemic postconditioning has demonstrated efficacy as an effective therapeutic intervention for acute ischemic stroke in experimental models by the neuroprotection against ischemia‐induced injury, regardless of the ground on which kinds of outcome measures including infarct size (Table 1), neurological scales (Table 2) and cell‐level tests (Table 3). Great treatment effects were often the outcome of a combination of many factors, which were classified into three aspects of animal models, postconditioning parameters, and measure time.

Factors for animal models were the selection of species and preparation of the stroke model, the latter being determined by the methods of ischemic induction and duration of ischemia. Of the 57 included studies with 224 experimental records, the vast majority used rodents including rats (number of studies, *N* = 46; number of experiments, *n* = 195) and mice (*N* = 10, *n* = 23), and only one study chose rhesus monkeys as experimental animals (*N* = 1, *n* = 6). In rodent studies, there was no statistical difference between species according to different outcome measures except for infarct size, but the conclusion needs to be further confirmed due to the relative lack of data in mice and an unbalanced distribution of treatment levels. While all measure indicators based on SMD consistently showed that different stroke preparation methods had no significant effect on the final comparison of treatment effects between the treatment and control group. In other words, the relative treatment effects of RIPostC in various types of stroke models were close. So, as long as reasonable and standardized operating procedures were followed, all the different models can be used for RIPostC research, among which the more common stroke model is MCAO 90–120 min (*N* = 31).

For the study using rhesus monkeys, only the data of infarct volume based on magnetic resonance imaging (MRI) 24 h after stroke were included in the meta‐analysis according to the criteria of data extraction and the result showed RIPostC did not reduce infarct size during the period, but the article clearly pointed out that serum levels of cardiac enzymes and endothelial injury marker decreased over the same time indicating that the RIPostC still had a certain effect on the ischemic monkey models in the hyperacute stage.^23^

On the other hand, the experts in the field have long called for and recommend that factors including age and gender should be taken into account when conducting research on remote ischemic conditioning,^39^ and studies have shown the effects of stroke rehabilitation and therapies vary by gender and age.^40^, ^41^, ^42^, ^43^ However, limited by inherent thinking and consideration of experimental convenience, the animal models for related research in the past decade were still dominated by adult male rodents. Of the 57 included studies, only three used females^32^, ^44^, ^45^ and two used mid‐aged or aged animals^37^, ^38^ (Table S1). Therefore, we regretted to exclude the two important factors of gender and age from our meta‐analysis due to their highly uneven distribution of data. Here, we also call on more peers to incorporate these factors into the experiments when engaging in relevant research.

In the postconditioning parameters, different numbers of treatments, treated body parts (hind limb or femoral artery), and number of sides almost had no statistical difference, which suggested that the multi‐visit, multi‐sided, and deliberate separation of femoral arteries for RIPostC had little value on acute ischemia, so the simpler noninvasive method of a single‐visit with an elastic bandage is enough to achieve the effect. And the parameters that really play a role in the treatment are conditioning time, number of cycles per treatment, duration of occlusion, and release per cycle. Firstly, the longer time point of conditioning is from the onset of stroke, the better the treatment effect is usually. This also provides a possible reason why Sun et al.,^30^ (conditioning at 3 or 6 h after the stroke of onset) is significantly higher in the Group B of neurological scales. But some studies conducting at 30 min after stroke onset^34^ or nearly 30 min after reperfusion onset^35^, ^36^ did not follow the trend. Secondly, three cycles per treatment were the most frequently used in experiments (*n* = 178) and appeared to have good effects (Figure 4, Table 1). Thirdly, the longer occlusion and release times per cycle resulted in better treatment effects, but the caveat is that there is no guarantee whether the prolonged duration works or not due to a few records of occlusion (*n* = 7) or release (*n* = 1) duration per cycle over 20 min (Figure 4, Table 1), and a duration of around 10 min are more recommended. In view of some above relatively less reliable and even contradictory conclusions, especially in conditioning time, comprehensive experimental studies on those contributing parameters are warranted in the future.

Measure time cannot inherently affect the treatment effect of RIPostC indeed, but the effect sizes might vary when measuring at different time points according to our experience and the analysis results (Tables 1 and 2) are in line with the expectations. In the study, measure time was grouped into ≤24 h (hyperacute stage), 48–72 h (namely 2–3 days), and 4–7 days (the two belong to the acute stage), >7 days (subacute stage).^46^ Based on infarct size, testing within 48–72 h showed better effects compared with that done at hyperacute stage and 4–7 days. On the other hand, previous studies by our group have revealed that loss of blood flow and nervous tissue damage to the ischemic area increased and reached the most at about 3 days after stroke in the rat models, then gradually recovered with recanalization and collateral revascularization.^42^, ^47^ The almost coincidence of the two time points indicates that RIPostC has a much prominent efficacy on severe injuries, more importantly, the measurement based on infarct size should be done in the early acute stage to more realistically reflect the therapeutic outcome. The results of scales, though, more conform to what we know that the performance of animal behaviors is getting better over time due to animals with certain self‐healing and adaptive capabilities. And the absence of differences at cell‐level tests may not only be limited by the small amount of data collected, but more likely that the stroke‐induced disturbances in brain homeostasis rapidly trigger the responses by neuroprotective mechanisms, and remain high at the cellular and molecular levels during the acute stage.^3^, ^4^, ^23^

As is well‐known to all, various levels of outcome measures have their respective advantages and disadvantages. In brief, cell‐level tests including autophagy and apoptosis are more accurate but only for a small portion of brain regions; neurological scales can evaluate the overall physical conditions but are relatively more general and imprecise; infarct size is between and the most used measurement in most studies at present. For the neurological scales, Longa 5‐point scale (SMD: 1.79 [1.21, 2.38]) was first designed and also the simplest, which is currently used by many studies as a criterion for animal enrolling after stroke induction,^24^ while mNSS 18‐point scale (SMD: 1.74 [1.31, 2.17]) might be a better alternative for subsequent behavioral evaluation, because of no statistical difference between them (*p* = 0.88) and the latter with more detailed assessment process.^26^

From the results of cell‐level tests, RIPostC has the ability to reduce normal cell death or apoptosis, and regulate autophagy to reduce I/R injury.^7^ Among the studies using autophagy as a measure, the majority showed that RIPostC provided neuroprotection by activating autophagy^34^, ^36^, ^48^, ^49^, ^50^; however, Chen et al.,^31^ illustrated that RIPostC protected the brain from damage by inhibiting autophagy. In addition, Wang et al.,^51^ (The study was also included in the analysis, but the measure of autophagy was performed based on the combination of RIPostC and ISIPostC, so this part of the data did not meet the criteria and was not in statistics.) reached similar conclusions to Chen et al., and further found that autophagy inhibitors also could reduce the injury from stroke. Conflicting conclusions from related studies suggest the neuroprotective mechanism of autophagy‐related pathways remains to be intensively and comprehensively studied in the future. It should also be pointed out that the effect sizes of two studies^52^, ^53^ based on H&E staining for the damaged‐neuron counting were significantly high, which might be the inaccuracy of the staining method for the statistics of damaged neurons in the brain after injury, and thus not recommended for cell quantitative counting, but acceptable for calculation of infarct size.^54^

Last but not least, the overall risk of bias for the included studies was low (89.5% low risk and 10.5% others according to the RoB 2 tool), but the meta‐analysis is subject to possible weaknesses limited by their risk of bias or study quality. Firstly, 18 studies (31.6%) were considered with “some concerns” on bias in the measurement of the outcome because they did not specify whether outcome evaluators knew that study animals received treatment.^13^ In general, people with the above information tend to unconsciously rate them in a biased way. Secondly, the included studies showed significant publication bias based on the Egger's and Begg's rank test no matter what the outcome measures were based on, which might lead to misestimate treatment effects without published negative data and needs to be wary of such survivorship bias. Thirdly, most of the studies (93.0%) did not estimate the sample size (one item of CAMARADES criteria) and only four studies (7.0%) did,^32^, ^37^, ^38^, ^55^ while an appropriate sample size is one of the most important factors in scientific research and statistical analysis, and should not be easily ignored.^56^

In conclusion, the postconditioning parameters that play a role in the final treatment effect of RIPostC are mainly conditioning time, number of cycles per treatment, duration of occlusion, and release per cycle, while numbers of treatments, treated body parts, and number of sides are less influential. According to the outcome measures, RIPostC can reduce the infarct size (most pronounced in the early acute stage), improve the behavioral ability of experimental animal models, reduce normal‐neuron death or apoptosis, and regulate the autophagy level. Due to the discrepancy in the trend of conditioning time and regulation mechanism of autophagy and little data in gender, age, and longer duration of conditioning, more in‐depth and systematic research is needed in these areas.

## FUNDING INFORMATION

This work was supported by the Natural Science Foundation of Zhejiang Province [grant number LY21H170002], Graduate Scientific Research Foundation of Hangzhou Dianzi University [grant number CXJJ2020047], and National Natural Science Foundation of China [grant number 31401008].

## CONFLICT OF INTEREST

The authors declare no conflicts of interest. The authors alone are responsible for the contents and writing of the article.

## AUTHOR CONTRIBUTION

K. Z. (Kezhou Liu) and Z. C. (Zhengting Cai) involved in the conception and design. Q. Z. (Quanwei Zhang), Y. C. (Yinuo Cheng), and M. Y. (Mengjie Yin) involved in the identification of studies. Q. Z. (Quanwei Zhang), Y. C. (Yinuo Cheng), J. H. (Jiatong He), and S. W. (Shaonong Wei) involved in the selection of studies. Q. Z. (Quanwei Zhang), Y. C. (Yinuo Cheng), J. H. (Jiatong He), and Z.C. (Zhengting Cai) involved in data extraction. Z. C. (Zhengting Cai) and Q. Z. (Quanwei Zhang) involved in the statistical analysis. Z. C. (Zhengting Cai) involved in writing—original draft. Z. C. (Zhengting Cai) and K. Z. (Kezhou Liu) involved in writing—review and editing. K. Z. (Kezhou Liu) involved in project administration.

## Supporting information

DocumentS1

DocumentS2

FigsureS2‐S7

TableS1‐S3

## Data Availability

The data that supports the findings of this study are available in the supplementary material of this article.
