# Supplementary material for: Determination of significant parameters in remote ischemic postconditioning for ischemic stroke in experimental models: A systematic review and meta‐analysis study
Source: CNS Neurosci Ther. 2022 Jul 27;28(10):1492–508. doi: 10.1111/cns.13925 (PMC9437239; doi:10.1111/cns.13925)
Supplement: Supplementary file 1 — DocumentS1 [file CNS-28-1492-s002.docx]

**Longa 5-point scale**

Longa 5-point scale [1], also named the neurological deficit score (NDS), was graded on a scale of 0-5. The higher the score, the more serious the neurological deficits (normal score = 0; maximal deficit score = 5).

While some studies claimed to use the scoring system from Bederson et al. (1986) [2]. After reviewing the original articles of the two scoring systems and comparing the differences with the follow-up research, we found that the later research was very confusing when using these two systems and citing articles. First, the original Bederson system reached 3 points at most, and follow-up studies added 4-point (no spontaneous walking and a depressed level of consciousness) [3] and 5-point (died) [4]; similarly, the original Longa system reached up to 4 points, and some studies added 5-point (died) [5]. Secondly, some articles said the Bederson system was used in their studies, but the actual scoring items listed were indeed the Longa system [5-6]. Fortunately, there was no remarkable difference between the two scoring systems, which also might be the reason why most subsequent studies tended to use a mixed version of the two systems. Here is unified into one scoring system named the Longa 5-point scale for the convenience of statistics. Hereby certify that, and the original scoring items of the two systems and the currently widely used revised version are both listed as below.

**Original Bederson scoring system [1]:**

- 0: no observable deficit;
- 1: forelimb flexion;
- 2: decreased resistance to lateral push (and forelimb flexion) without circling;
- 3: same behavior as grade 2, with circling;
- 0: no neurologic deficit.

**Original Longa scoring system [2]:**

- 0: no neurologic deficit;
- 1: a mild focal neurologic deficit (failure to extend left forepaw fully);
- 2: a moderate focal neurologic deficit (circling to the left);
- 3: a severe focal deficit (falling to the left);
- 4: no spontaneous walking and a depressed level of consciousness;

**Revised mixed version [4, 6]:**

- 0: no neurologic deficit;
- 1: a mild focal neurologic deficit (forelimb flexion, namely failure to extend left/right forepaw fully);
- 2: a moderate focal neurologic deficit (circling to the left/right);
- 3: a severe focal deficit (falling to the left/right);
- 4: no spontaneous walking and a depressed level of consciousness;
- 5: died.
- Note: the direction in criteria (left/right) was the contralateral side to cerebral artery occlusion; the maximum score of 5 would not appear due to the included animal models showing no death.

**Belayev 12-point scale**

Belayev 12-point scale [7] was graded on a scale of 0–12 (normal score = 0; maximal deficit score = 12). The details of scoring criteria in Table 1.

**Table 1. Neurological Evaluation by Belayev 12-point scale ^[7]^**

| **Item** | **Normal Score** | **Deficit Score** |
| --- | --- | --- |
| Postural reflex (“hang test”) ^*^ | 0 | 2 |
| Placing test (performed on each side) ^#^ |  |  |
| Visual placing | 0 | 2 |
| Forward | 0 | 2 |
| Sideways |  |  |
| Tactile placing |  |  |
| Dorsal surface of paw | 0 | 2 |
| Lateral surface of paw | 0 | 2 |
| Proprioceptive placing | 0 | 2 |
| Total score | 0 | 12 |

* scored by: 0, no observable deficit; 1, limb flexion during hang test; 2, deficit on lateral push.

# scored by: 0, complete immediate placing; 1, incomplete and/or delayed placing (<2s); 2, absence of placing.

**Ladder rung walking test**

The apparatus of Ladder rung walking test [8-9] was a nearly 1m long horizontal runway with rungs placed in the irregular pattern and the distance between rungs varying from 7 to 14mm. The test was usually carried out by multiple sessions and in each session the number of errors and steps of the affected forelimb and hindlimb was counted. The final score was determined by the average of foot-fault numbers. Higher score represented worse deficit, and score of zero indicated no observable deficit.

**mNSS 18-point scale**

Modified neurological severity scores (mNSS) 18-point scale [10] was graded on a scale of 0–18 (normal score = 0; maximal deficit score = 18). The details of scoring criteria in Table 2.

**Table 2. modified Neurological Severity Scores (mNSS)** **^[10]^**

| **Items** | **Points** |
| --- | --- |
| **Motor tests**  **Raising rat by the tail**   - 1: Flexion of forelimb - 1: Flexion of hindlimb - 1: Head moved＞10° to vertical axis within 30 s | 3 |
| **Placing rat on the floor (normal=0; maximum=3)**   - 0: Normal walk - 1: Inability to walk straight - 2: Circling toward the paretic side - 3: Fall down to the paretic side | 3 |
| **Sensory tests**   - 1: Placing test (visual and tactile test) - 1: Proprioceptive test (deep sensation, pushing the paw against the table edge to stimulate limb muscles) | 2 |
| **Beam balance tests (normal=0; maximum=6)**   - 0: Balances with steady posture - 1: Grasps side of beam - 2: Hugs the beam and one limb falls down from the beam - 3: Hugs the beam and two limbs fall down from the beam, or spins on beam (＞60 s) - 4: Attempts to balance on the beam but falls off (＞40 s) - 5: Attempts to balance on the beam but falls off (＞20 s) - 6: Falls off: No attempt to balance or hang on to the beam (＜20 s) | 6 |
| **Reflexes absent and abnormal movements**   - 1: Pinna reflex (head shake when touching the auditory meatus) - 1: Corneal reflex (eye blink when lightly touching the cornea with cotton) - 1: Startle reflex (motor response to a brief noise from snapping a clipboard paper) - 1: Seizures, myoclonus, myodystony | 4 |
| **Maximum points** | 18 |

One point is given for the inability to perform tasks or lack of the tested reflex

**Garcia 18-point scale**

Garcia 18-point scale [11] was graded on a scale of 3–18 (normal score = 18; maximal deficit score = 3). The details of scoring criteria in Table 3.

**Table 3. Neurological Evaluation by Garcia 12-point scale ^[11]^**

| **Test** | **Score** | | | |
| --- | --- | --- | --- | --- |
|  | **0** | **1** | **2** | **3** |
| **Spontaneous activity (in cage for 5 min)** | No movement | Barely moves | Moves but does not approach at least three sides of cage | Moves and approaches at least three sides of cage |
| **Symmetry of movements (four limbs)** | Left side: no movement | Left side: slight movement | Left side: moves slowly | Both sides: move symmetrically |
| **Symmetry of forelimbs (outstretching while held by tail)** | Left side: no movement, no outreaching | Left side: slight movement to outreach | Left side: moves and outreaches less than right side | Symmetrical outreach |
| **Climbing wall of wire cage** | / | Fails to climb | Left side is weak | Normal climbing |
| **Reaction to touch on either side of trunk** | / | No response on left side | Weak response on left side | Symmetrical response |
| **Response to vibrissae touch** | / | No response on left side | Weak response on left side | Symmetrical response |

**Reference**

[1] Longa EZ, Weinstein PR, Carlson S, et al. Reversible middle cerebral artery occlusion without craniectomy in rats. Stroke, 1989, 20(1): 84-91.

[2] Bederson J B, Pitts L H, Tsuji M, et al. Rat middle cerebral artery occlusion: evaluation of the model and development of a neurologic examination[J]. stroke, 1986, 17(3): 472-476.

[3] Bonova P, Gottlieb M. Blood as the carrier of ischemic tolerance in rat brain[J]. Journal of neuroscience research, 2015, 93(8): 1250-1257.

[4] Hoda M N, Siddiqui S, Herberg S, et al. Remote ischemic perconditioning is effective alone and in combination with intravenous tissue-type plasminogen activator in murine model of embolic stroke[J]. Stroke, 2012, 43(10): 2794-2799.

[5] Kitagawa K, Saitoh M, Ishizuka K, et al. Remote limb ischemic conditioning during cerebral ischemia reduces infarct size through enhanced collateral circulation in murine focal cerebral ischemia[J]. Journal of Stroke and Cerebrovascular Diseases, 2018, 27(4): 831-838.

[6] Yang G, Chan P H, Chen J, et al. Human copper-zinc superoxide dismutase transgenic mice are highly resistant to reperfusion injury after focal cerebral ischemia[J]. Stroke, 1994, 25(1): 165-170.

[7] Belayev L, Alonso O F, Busto R, et al. Middle cerebral artery occlusion in the rat by intraluminal suture: neurological and pathological evaluation of an improved model[J]. Stroke, 1996, 27(9): 1616-1623.

[8] Metz G A, Whishaw I Q. Cortical and subcortical lesions impair skilled walking in the ladder rung walking test: a new task to evaluate fore-and hindlimb stepping, placing, and co-ordination[J]. Journal of neuroscience methods, 2002, 115(2): 169-179.

[9] Sun F, Xie L, Mao X O, et al. Effect of a contralateral lesion on neurological recovery from stroke in rats[J]. Restorative neurology and neuroscience, 2012, 30(6): 491-495.

[10] Chen J, Li Y, Wang L, et al. Therapeutic benefit of intravenous administration of bone marrow stromal cells after cerebral ischemia in rats[J]. Stroke, 2001, 32(4): 1005-1011.

[11] Garcia J H, Wagner S, Liu K F, et al. Neurological deficit and extent of neuronal necrosis attributable to middle cerebral artery occlusion in rats: statistical validation[J]. Stroke, 1995, 26(4): 627-635.
