# Supplementary material for: Determination of significant parameters in remote ischemic postconditioning for ischemic stroke in experimental models: A systematic review and meta‐analysis study
Source: CNS Neurosci Ther. 2022 Jul 27;28(10):1492–508. doi: 10.1111/cns.13925 (PMC9437239; doi:10.1111/cns.13925)
Supplement: Supplementary file 2 — DocumentS2 [file CNS-28-1492-s003.docx]

**Data preprocessing**

**The condition of non-zero standard deviation (SD):** The algorithm of standardized mean difference (SMD) requires a non-zero SD for each measurement, thereout it would be approximated by the mean of the SD of non-zero measurements in the same study when the SD of a measure is zero.

**Infarct area:** Most of studies calculated the proportion of infarct area to half of brain, therefore the mean and SD of infarct size for those using the percentage of whole brain would be multiplied by 2. And considering widely different measurement scales of infarct size, the data of each infarct size was normalized, i.e., both of mean and SD in control group and treatment group were divided by the mean in control group.

**Neurological scales and cell-level tests:** The outcome of various neurological scales and cell-level tests always fell into one of two categories, with either a higher evaluation value indicating more severe deficit or a higher evaluation value indicating more normalcy. For the unified representation of numerical values, the mean and SD values of outcome in the second category were preprocessed as follows.

$\tilde{Mean}=\frac{{Max}_{Mean}-Mean}{{Max}_{Mean}-{Min}_{Mean}}$ , $\tilde{SD}=\frac{SD}{{Max}_{Mean}-{Min}_{Mean}}$

where $Mean$ and $SD$ are the original mean and SD values of outcome in one experimental record, $\tilde{Mean}$ and $\tilde{SD}$ are the preprocessed mean and SD values, ${Min}_{Mean}$ and ${Max}_{Mean}$ are the minimal and maximal values of outcomes among one group.

**Notably, normalization and standardization cannot change the final effect size from the algorithm of SMD.**

**Grouping**

The values of same factor used in different studies vary with the purpose of research. In order to avoid just a few experimental records in some groups, certain factors were regrouped based on our past experience and data distribution at first (Figure 2). For the factor of stroke model, transient ischemia had subgroups of middle cerebral artery occlusion (MCAO) 30-60 min, MCAO 90-100min, MCAO 120min and MCAO 180min, while embolic MCAO (eMCAO), permanent MCAO (pMCAO) and bilateral common carotid artery occlusion with distal MCAO (BBCAO & dMCAO) belonged to permanent ischemia, and global ischemia only consisted of global ischemic four-vessel occlusion (4-VO). In terms of time-related factors, grouping of conditioning time (single-visit treatment) requires additional clarification, as shown in Figure S1. Data extracted from records based on various neurological scales and cell-level tests cannot be directly combined into one group for analysis because of different experimental principles and scoring priorities, and they would be merged into as few groups as possible and meet the criterion that there is no statistical difference within groups but significant differences between groups


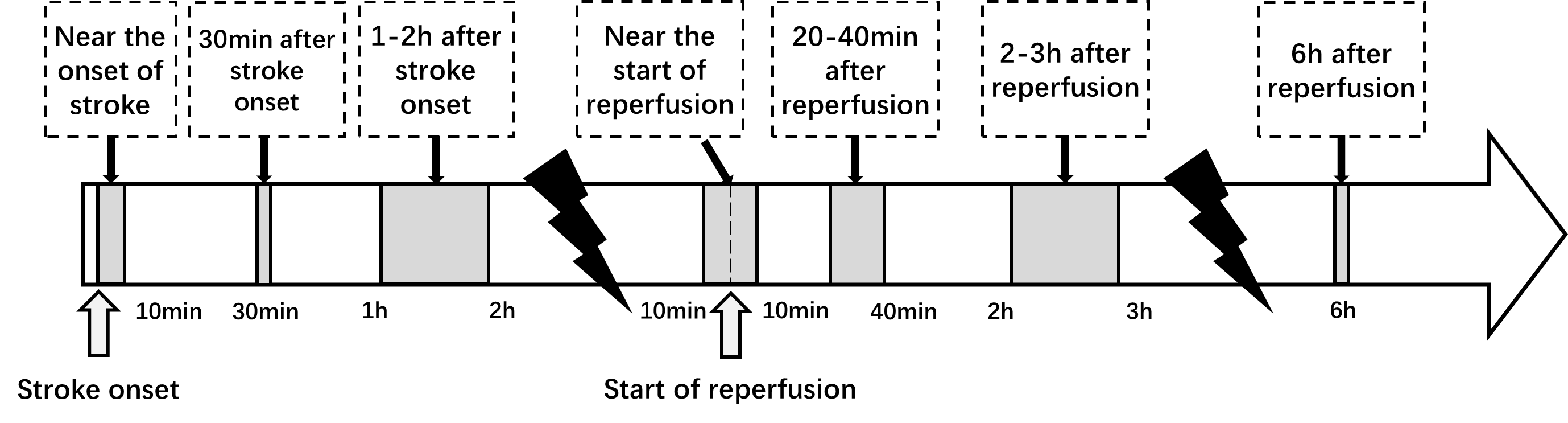


**Figure S1. Conditioning time (single-visit treatment) grouping.**

The group of “Near the onset of stroke” represents the conditioning time within ten minutes after the onset of stroke, and the group of “Near the start of reperfusion” represents the conditioning time within ten minutes before and after the start of reperfusion.

min, minutes; h, hours; d, days.
