# Supplementary material for: Determination of significant parameters in remote ischemic postconditioning for ischemic stroke in experimental models: A systematic review and meta‐analysis study
Source: CNS Neurosci Ther. 2022 Jul 27;28(10):1492–508. doi: 10.1111/cns.13925 (PMC9437239; doi:10.1111/cns.13925)
Supplement: Supplementary file 3 — FigsureS2‐S7 [file CNS-28-1492-s001.docx]

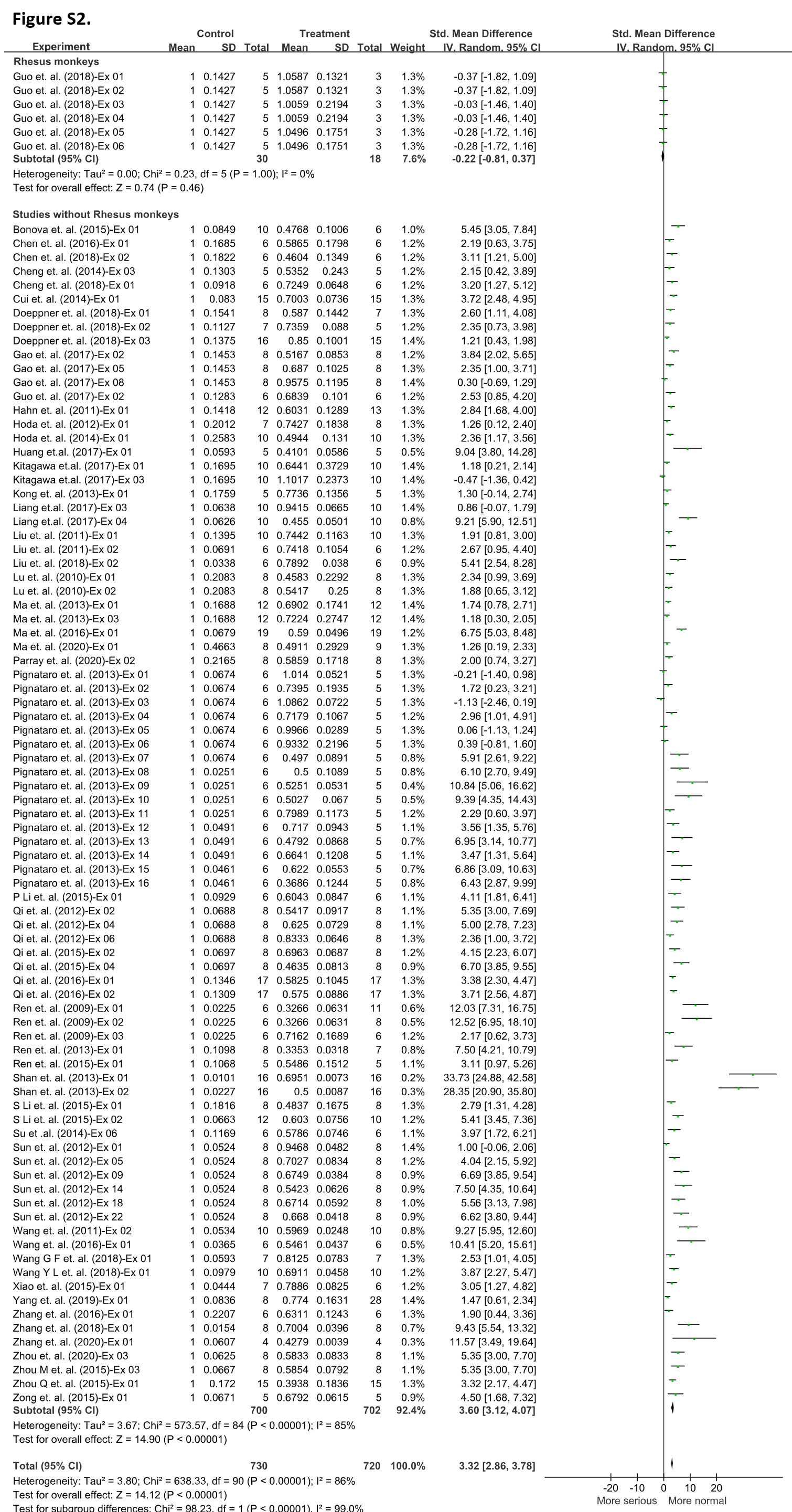


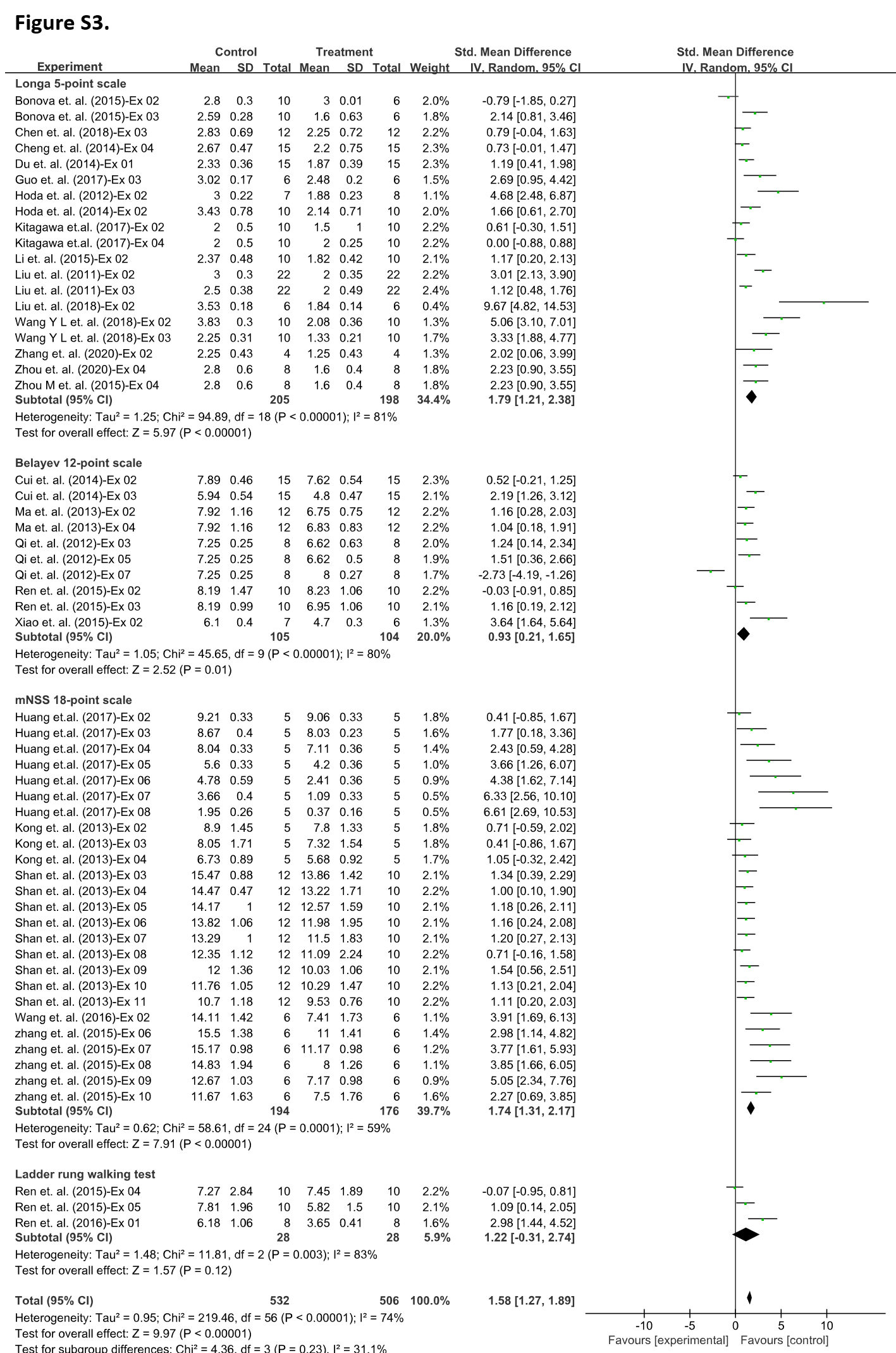


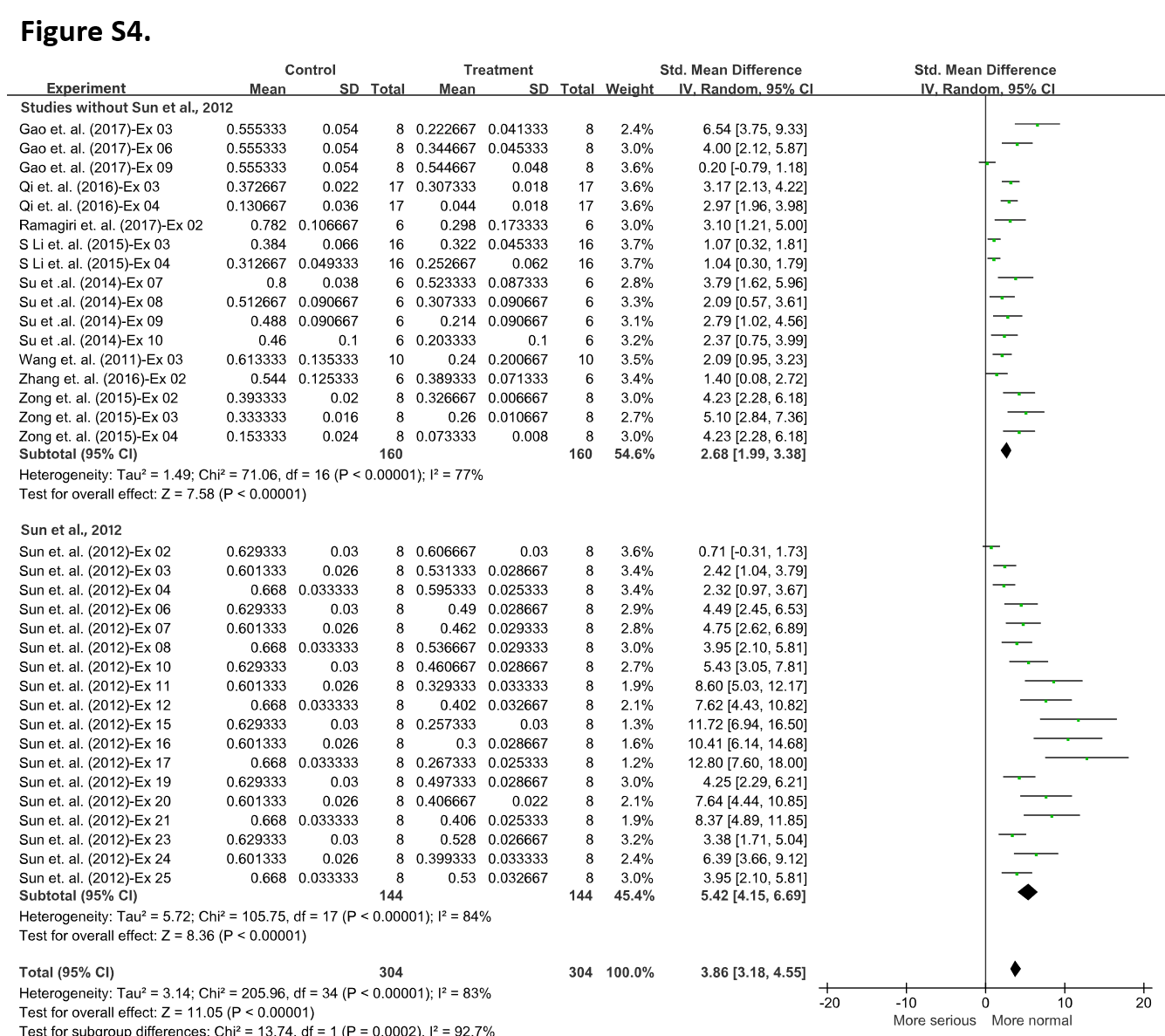


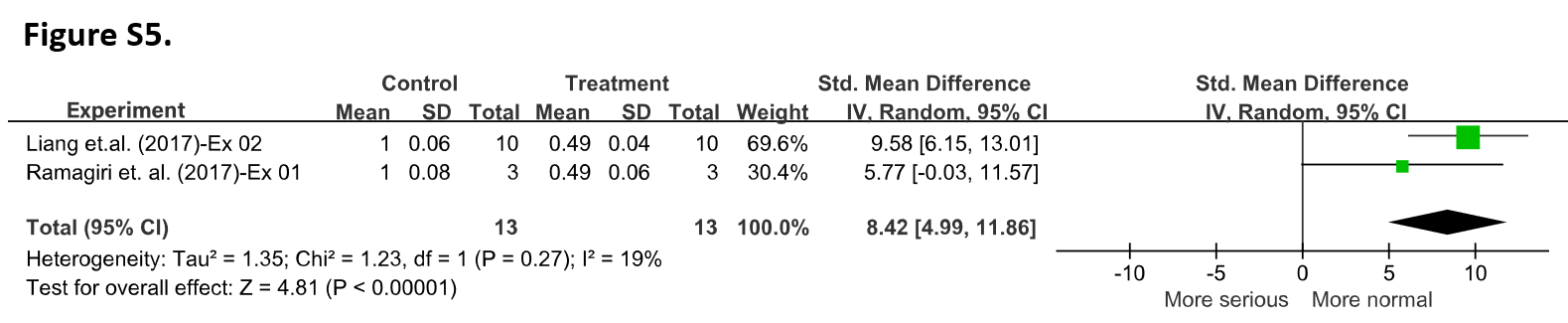


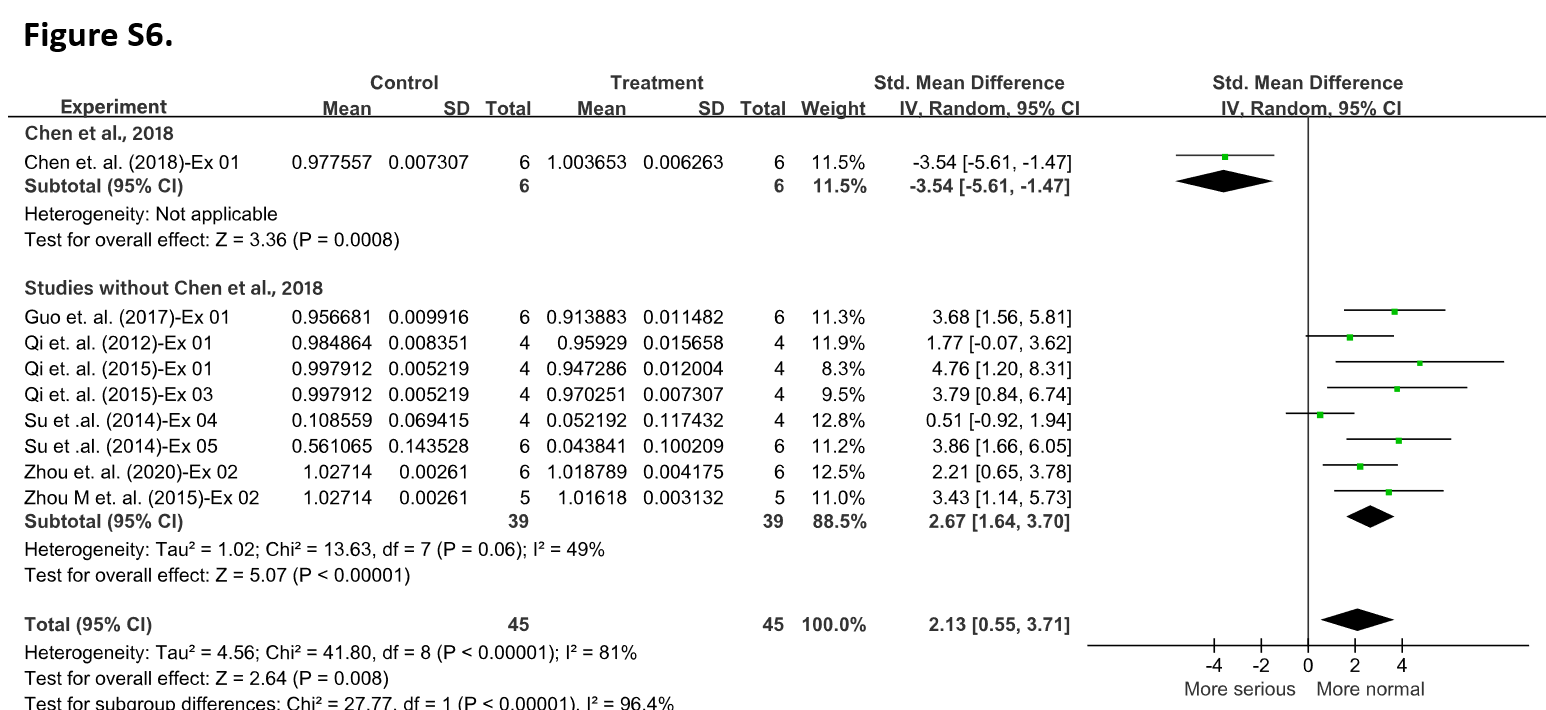


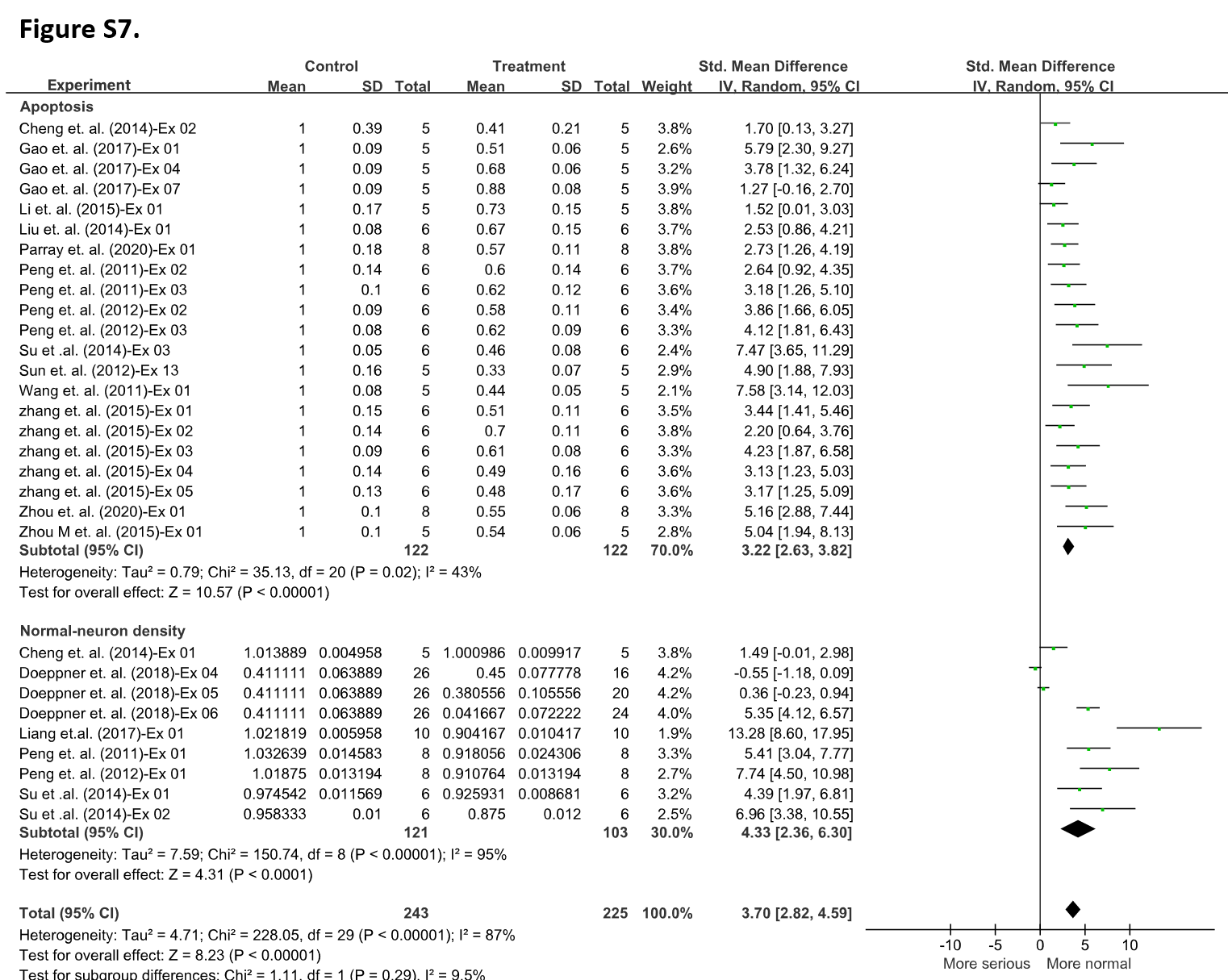


**Figure S2-7. Forest plots of each group of all outcome measures including infarct size (Figure S2.), Group A (Figure S3.) and Group B (Figure S4.) in neurological scales, Group C (Figure S5.), Group D (Figure S6.) and Group E (Figure S7.) in cell-level tests.**

All of forest plots were generated in the software of RevMan 5.4.1 and then modified according to our study’ purpose. To facilitate analysis and comparison, some data of mean and SD in specific groups were standardized and normalized, which would not change the final treatment effect presented by standardized mean difference (SMD) due to the property of SMD algorithm.

SD, standard deviation; Std, standardized; IV, inverse-variance method.
